# Wide-Field-of-View Longwave Camera for the Characterization of the Earth’s Outgoing Longwave Radiation

**DOI:** 10.3390/s21134444

**Published:** 2021-06-29

**Authors:** Luca Schifano, Lien Smeesters, Francis Berghmans, Steven Dewitte

**Affiliations:** 1 Brussels Photonics (B-PHOT), Applied Physics and Photonics Department, Vrije Universiteit Brussel, Pleinlaan 2, 1050 Brussels, Belgium; lsmeeste@b-phot.org (L.S.); francis.berghmans@vub.be (F.B.); 2 Royal Meteorological Institute of Belgium, Avenue Circulaire 3, 1180 Brussels, Belgium; steven.dewitte@meteo.be; 3 Flanders Make, Pleinlaan 2, 1050 Brussels, Belgium

**Keywords:** Earth Radiation Budget, Earth Energy Imbalance, Outgoing Longwave Radiation, wide field of view, space instrumentation, radiative transfer simulations, aspherical optical design, refractive imaging system

## Abstract

The measurement of the Earth’s Outgoing Longwave Radiation plays a key role in climate change monitoring. This measurement requires a compact wide-field-of-view camera, covering the 8–14 µm wavelength range, which is not commercially available. Therefore, we present a novel thermal wide-field-of-view camera optimized for space applications, featuring a field of view of 140° to image the Earth from limb to limb, while enabling a high spatial resolution of 4.455 km at nadir. Our cost-effective design comprises three germanium lenses, of which only one has a single aspherical surface. It delivers a very good image quality, as shown by the nearly-diffraction-limited performance. Radiative transfer simulations indicate excellent performance of our camera design, enabling an estimate of the broadband Outgoing Longwave Radiation with a random relative error of 4.8%.

## 1. Introduction

In our pursuit to better understand Earth’s changing climate, the monitoring of the Earth’s radiation budget (ERB) is of major importance [1]. This budget quantifies the radiative energy fluxes at the top-of-atmosphere (TOA), which can best be monitored from space. The state-of-the-art ERB measurements have been provided so far by NASA’s Clouds and the Earth’s Radiant Energy System (CERES) program [2,3]. They feature a spatial resolution equal to 20 km at nadir for the CERES instrument on-board of the Terra and Aqua satellites, and 10 km for the CERES instrument on-board of the Tropical Rainfall Measuring Mission (TRMM). The latter, as a precessing satellite, samples all solar illumination configurations, which makes it well suited for the development of Angular Distribution Models (ADM) [4]. As the field of view (FOV) of CERES is narrow (1.3° × 2.6°), the full viewing angle coverage is obtained by the CERES rotating azimuth scan capability. Scene identification is provided by a multispectral imager, the MODIS imager, flying on the same satellites as the CERES instrument, e.g., on the Terra and Aqua satellites [5].

To improve this monitoring, we propose a novel space mission, with a payload that combines several wide-field-of-view (WFOV) instruments, which allow observing the Earth from limb to limb. With this WFOV, no scanning elements are required, which eases the mechanical integration. In addition, our design fits within 4 dm^3^ units of a CubeSat, forming a compact and relatively low-cost payload, suitable for integration on nano- or micro-satellites. Such small satellites can be used to supplement CERES and its follow-on mission Libera—the space mission recently selected in the framework of the Earth Venture Continuity [6,7]—e.g., for improving the sampling of the diurnal cycle. In this context, our design is particularly relevant since currently no follow-on mission for the sampling of the ERB from the morning orbit is foreseen after the end of life of the CERES instrument on the Terra satellite, which is expected around 2026.

The first instrument of our payload is a WFOV radiometer [8] that aims to measure the total radiation emitted by both the Sun and the Earth, with an estimated accuracy of 0.44 W/m^2^. The ability to measure those quantities with the same instrument decreases the calibration errors, and leads to an accurate measurement of the Earth’s Energy Imbalance (EEI), which is the key parameter that drives the current climate change [9,10,11]. Combining this radiometer with WFOV cameras allows increasing the spatial resolution and enables identifying scenes. In addition, the use of cameras operating in different wavelengths regions allows distinguishing between the Reflected Solar Radiation (RSR), measured by a shortwave (SW, [400–1100] nm) camera [12], and the Outgoing Longwave Radiation (OLR), measured by a longwave (LW, [8–14] µm) camera.

This paper deals with the WFOV LW camera that aims to characterize the OLR with a maximum relative error of 5%, and where we target the optical design to achieve a nadir spatial resolution of minimally 5 km. To the best of our knowledge, there is no commercial off-the-shelf (COTS) compact camera that operates in the [8–14] µm wavelength range and that features a FOV of 140° for the characterization of the OLR. State-of-the-art thermal cameras include either imaging designs operating in this wavelength range but with a smaller FOV (51° × 40°) [13], or cameras with the required FOV but in another and smaller wavelength range ([14–16] µm) [14], or systems that both operate in a different wavelength range and feature a smaller FOV, e.g., in [15]. Considering the required wavelength range, the maximal FOV that is currently published is obtained with a catadioptric objective and equals to 125° × 96° [16], which is still significantly smaller than our targeted value of 140° (circular full FOV).

Our manuscript is structured as follows: the first part (Section 2) is dedicated to the optical system design of the LW camera, while the second part (Section 3) is devoted to the estimate of the broadband OLR using this LW camera. The latter also reports on its performance based on radiative transfer simulations. Section 4 discusses our results as well as prospects for future work, and Section 5 closes this paper with a summary.

## 2. Optical System Design

This section focuses on the optical system design of the LW camera. Section 2.1 discusses the technical requirements and constraints that drive the optical design, while Section 2.2 presents the optical camera system, as well as an evaluation of the image quality, based on spot sizes, contrast and aberrations.

### 2.1. Scientific and Technical Requirements

The optical system design must account for the following set of requirements.

(1) The Earth should be seen from limb to limb, from a nominal satellite altitude of 700 km.
(2) The camera should enable scene identification.
(3) The camera should measure LW radiation, allowing to reconstruct the OLR on a stand-alone basis with an error of maximally 5%, as stated in [8].
(4) At nadir, the camera should have a resolution of 5 km or better.
(5) The volume of the camera (optics and detector) should fit within 1 CubseSat Unit (1U).
(6) The optical system should be designed with a minimal amount of optical elements and optical materials, in order to limit the cost and ease the fabrication.

To observe the Earth from limb to limb from an altitude of 700 km, the full FOV should be minimally 127° = 2 × 63.5°. Taking a margin for the altitude and pointing errors into account, we target a slightly larger FOV, of the order of 2 × 70°. This margin also allows for some additional design freedom and enables reducing the aberrations and/or relaxing the tolerances in the final design stage, by reverting to a slightly smaller FOV. Regarding the detector, we favor the use of a COTS uncooled microbolomoter array that is sensitive in the range from 8 to 14 µm. We selected the ULIS/Lynred Pico1024Gen2 detector, comprising 1024 × 768 pixels, with a pixel pitch of 17 µm. To image the Earth from limb to limb, we use a circular area on the detector with a radius of 6.528 mm.

### 2.2. Optical Design of the LW Camera

**Figure 1:**
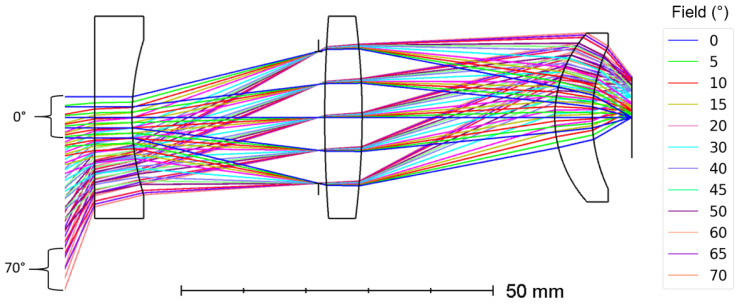
Optical design of the LW camera. The total axial length equals 86.12 mm. The system consists of 3 singlet lenses. The aperture stop is situated between the first two lenses. The circular image on the detector has a radius of 6.528 mm. The different colors correspond to the different fields between 0° and 70°.

The optical design of the longwave camera is refractive and makes use of three germanium lenses (Figure 1). The back surface of the second lens is an asphere described up to the 8th order aspheric term. Germanium lenses can be found in the SCHOTT^®^ catalog [17] and are suited for space applications. They have a high refractive index (4.0 between 8 and 14 µm), allowing us to bend the incoming rays efficiently with a minimum number of optical elements (Table 1). Note that the amount of elements is intentionally limited to reduce the cost of the optical design and to fit the full system within 1U. The stop aperture has a diameter of 21 mm and is placed just before the second lens, such that each field fills the full aperture. Also, the *High-Yield manufacturing*-feature of Zemax OpticStudio^®^ [18] has been used to improve the manufacturing feasibility of our design. To optimize our optical system, we start with the spot size as our merit function. Once the design is near-diffraction-limited, we further evaluate the image quality by considering the Modulation Transfer Function (MTF). Our optical design (Figure 1) is obtained after iterative optimization of the lens parameters, yielding an optimized performance, while taking the above requirements and constraints into account.

**Figure 2:**
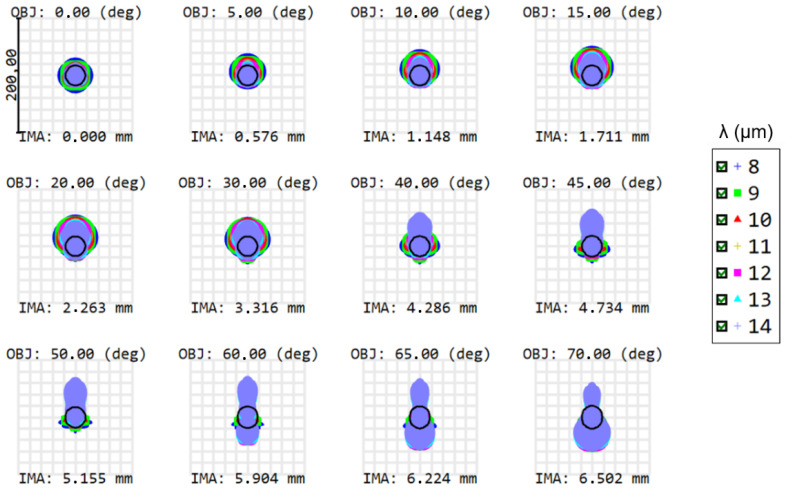
Spot sizes for fields between 0° and 70°, for all wavelengths between 8 and 14 µm (as visualized by the different colors in each spot plot). The Airy disk radii (black circles) equal 17.28 µm. Based on the spots, the system shows a good image quality. OBJ (in degrees) defines the object field and IMA (in mm) defines the image height of the centroid on the detector. This figure indicates that the best performance is obtained on-axis, while off-axis spots show less favorable performance.

The full FOV is circular and equals 140° (Figure 1), which is sufficient to cover the Earth from limb to limb while foreseeing a margin for pointing errors. To evaluate the performance of the camera system, we consider the spot diagrams shown in Figure 2. The spot size is simulated for different fields between 0° and 70°, corresponding to the fields presented in Figure 1. In Figure 2, the black circles correspond to the Airy disks and the different colors represent the different wavelengths. When optimizing the optical design, the aim is to match the RMS spot size with the Airy disk, to obtain a near-diffraction-limited optical design, where the Airy disk is calculated with the Nyquist criterion.

The main design challenge is the wide full FOV of 140°. In addition to the fact that this imposes a major challenge to correct the aberration at all fields, a low f-number (ratio *f*/*D*) of 1.0 is required to match the Airy disk size with the pixel size. Nevertheless, it can be managed using a single aspheric surface (Table 1). As can be seen in (Table 2), the obtained spot sizes are close to the Airy disk sizes, for all fields and wavelengths.

**Figure 3:**
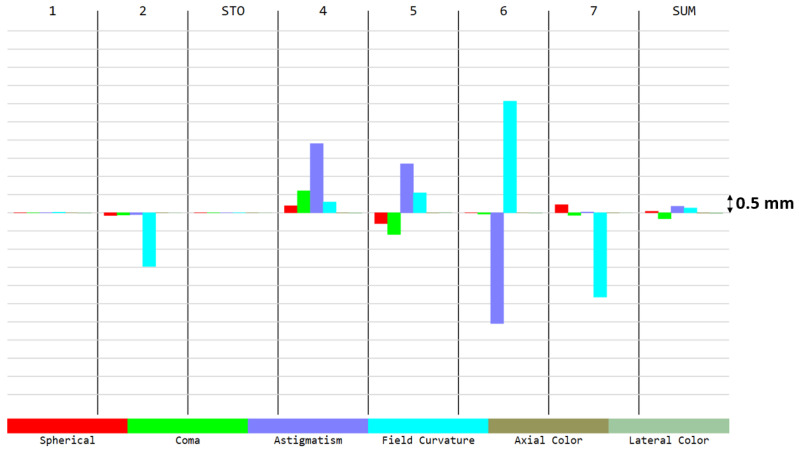
Seidel aberrations and (axial and lateral) chromatic aberrations, except for distortion. The aberrations are given for each surface involved in the optical system (1 and 2 for the first lens, 3 = STO i.e., the stop aperture, 4 and 5 for the second lens, 6 and 7 for the third lens). The SUM represents the sum of these aberrations at the image plane. All these aberrations are corrected. The same aberration contributions were observed for all wavelengths between 8 and 14 µm.

**Figure 4:**
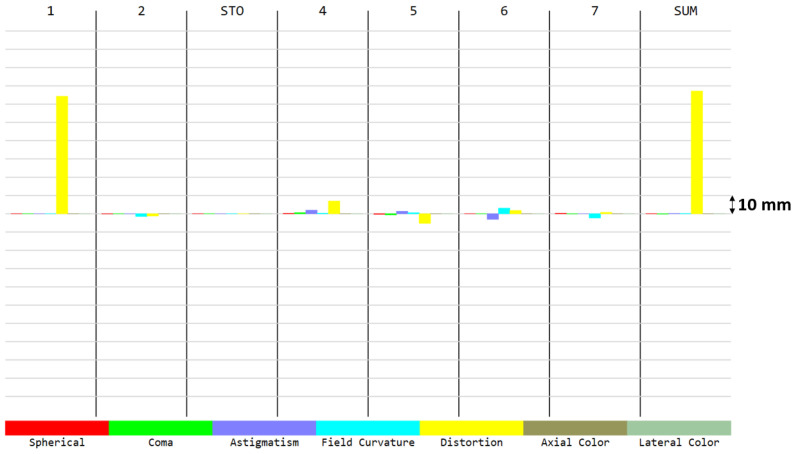
Five Seidel aberrations and (axial and lateral) chromatic aberrations. The aberrations are given for each surface involved in the optical system (1 and 2 for the first lens, 3 = STO i.e., the stop aperture, 4 and 5 for the second lens, 6 and 7 for the third lens). The SUM represents the sum of these aberrations at the image plane. The main aberration is the barrel distortion, mainly induced by Surface 1, i.e., the front surface of the first lens. The barrel distortion is further detailed in Figure 5. The same aberration contributions were observed for all wavelengths between 8 and 14 µm.

**Figure 5:**
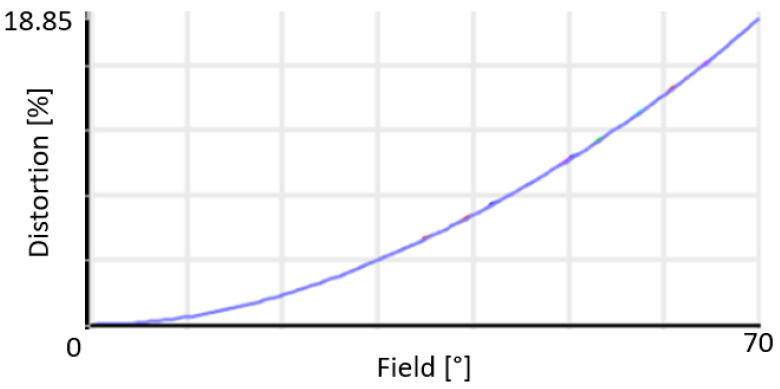
Barrel distortion is the main aberration present in the optical design, due to the wide field of view. Distortion is maximal at 70°, where it equals 18.85%. In this graph, the vertical axis expresses the distortion in %, and the horizontal axis gives the half FOV in degrees.

To better understand the contributions for each aberration present in the spot diagram (Figure 2), we can have a look at the Seidel diagram (Figure 3 and Figure 4), which quantifies the common aberrations in the optical system (spherical, coma, astigmatism, field curvature, distortion, axial color, lateral color) [19]. Distortion generally increases with the field, and therefore it is a common aberration in WFOV imaging systems. The front surface of the first lens contributes the most to distortion. However, there is no particular requirement on this aberration since it can be measured during pre-flight characterization and can be taken into account during the in-flight processing. When considering all aberrations except distortion, positive aberrations of one lens surface are compensated by negative aberrations of another lens, minimizing the total aberrations at the detector (Figure 3). Consequently, barrel distortion remains the main aberration at the detector (Figure 4). A quantitative view on the barrel distortion is given in Figure 5, showing that distortion is maximal at 70°, where it equals 18.85%.

**Figure 6:**
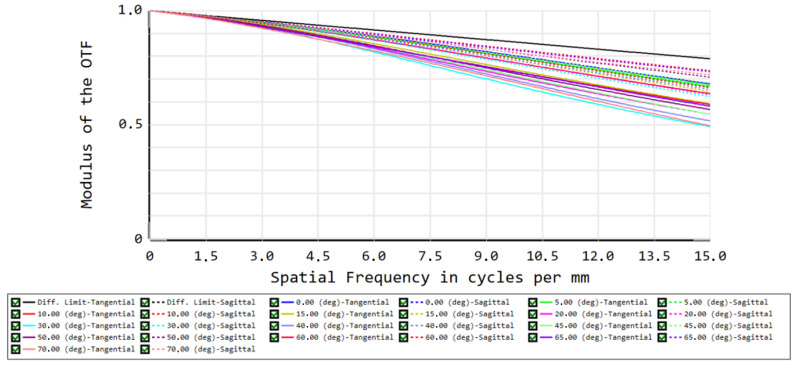
MTF ≥ 0.5 at 15 cycles/mm. The top black line corresponds to the diffraction limit, and the colors correspond to the different fields, similar to those in Figure 1. Full lines and dashed lines correspond to tangential and sagittal planes, respectively.

The image quality is subsequently evaluated by simulation of the MTF, which quantifies the spatial constrast. It results in a polychromatic (between 8 and 14 µm) diffraction MTF ≥ 0.5 at 15 cycles/mm (Figure 6). Considering a pixel pitch of 17 µm, this MTF indicates a good performance, satisfying the Nyquist criterion, avoiding undersampling of the image spatial variability and exaggerated blurring of the image.

## 3. Remote Sensing of the Outgoing Longwave Radiation

This section focuses on the estimate of the OLR by our developed longwave camera, and describes both the methods and results. Our camera operates between 8 and 14 µm, which is a narrow range of wavelengths compared to the spectral bandwidth of the OLR that ranges from 3 to 100 µm. The goal of our simulations is to estimate this broadband radiation (the OLR), described by what we call a *broadband temperature*, with a narrowband radiation (our estimation of the OLR using our LW camera) described by a *narrowband temperature*. The relations between the quantities pertaining to the radiation and the temperature are obtained by applying Planck’s radiation law and Stefan-Boltzmann’s law (cf. below). To this end, we perform radiative transfer simulations in libRadtran [20], in which different atmospheric conditions can be defined (atmospheric temperature and gas profiles, aerosols, clouds, surface properties). For this study, we choose the widely-used six clear-sky standard atmospheres from Anderson et al. [21]: U.S. Standard, Tropical, Midlatitude Summer, Midlatitude Winter, Subarctic Summer, and Subarctic Winter. For some of these standard atmospheres (U.S. Standard, Midlatitude Winter, and Subarctic Summer), we define the clouds in three different cases: water clouds, thin ice clouds, and thick ice clouds. This leads to a total of 15 different scenes, that are summarized in Table 3. The simulated data is subsequently used to assess the performance of our LW camera.

**Figure 7:**
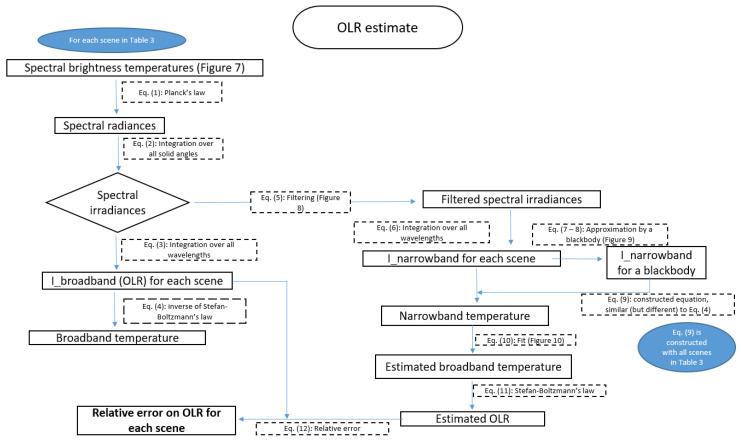
Flowchart summarizing the approach to estimate the Outgoing Longwave Radiation.

The OLR is estimated for each scene using the following approach (Figure 7):

- Simulations of the spectral brightness temperatures, in libRadtran;
- Computation of the spectral irradiances;
- Computation of the broadband temperature;
- Computation of the narrowband temperature;
- Fit of the broadband temperature as function of the narrowband temperature;
- Estimation of the OLR and calculation of the error on the OLR.

### 3.1. Simulations of Spectral Brightness Temperatures

**Figure 8:**
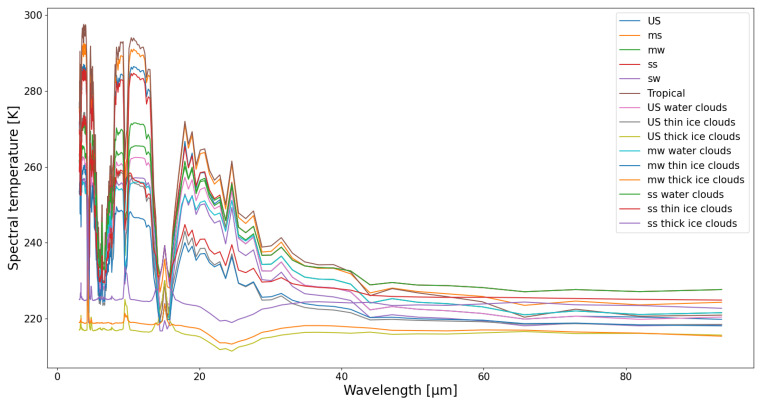
Spectral brightness temperature as function of wavelength for simulated scenes, listed in Table 3.

For each of the 15 scenes, the spectral brightness temperatures $T_{\lambda }$ are generated in the wavelength range from 3 µm to 100 µm (Figure 8).

### 3.2. Computation of the Spectral Irradiances

Each spectral brightness temperature $T_{\lambda }$ (with λ the wavelength) is converted into spectral radiances L(λ) by using Planck’s law [22], in Equation (1), with $h=6.62607015\times 10^{-34}$ J.s the Planck’s constant, c=299792458 m/s the speed of light, and $k=1.380649\times 10^{-23}$ J/K the Boltzmann’s constant. These spectral radiances L(λ) are then converted into spectral irradiances I(λ) by integration over all solid angles Ω, using Equation (2).

(1) $$L\left(\lambda \right)=\frac{2hc^{2}}{\lambda^{5}}\frac{1}{e^{\frac{hc}{\lambda kT_{\lambda }}}-1}$$

(2) $$I\left(\lambda \right)=\int_{\Omega }L\left(\lambda \right)\;d\Omega =\pi L\left(\lambda \right)$$

### 3.3. Computation of the Broadband Temperatures

From these spectral irradiances I(λ), we can derive the broadband irradiance (the OLR) associated with each scene. By integrating the spectral irradiances over the wavelengths using Equation (3), we obtain the broadband irradiance, from which the broadband temperature $T_{\mathrm{broadband}}$ can be derived, using the inverse of Stefan-Boltzmann’s law [22], given by Equation (4).

(3) $$\mathrm{OLR}=\int_{\lambda }I\left(\lambda \right)d\lambda$$

(4) $$T_{\mathrm{broadband}}=\sqrt[{4}]{\sigma \;\mathrm{OLR}}$$

where $\sigma \approx 5.670374\times 10^{-8}$ Wm^−2^K^−4^ is the Stefan-Boltzmann’s constant.

### 3.4. Computation of the Narrowband Temperatures

**Figure 9:**
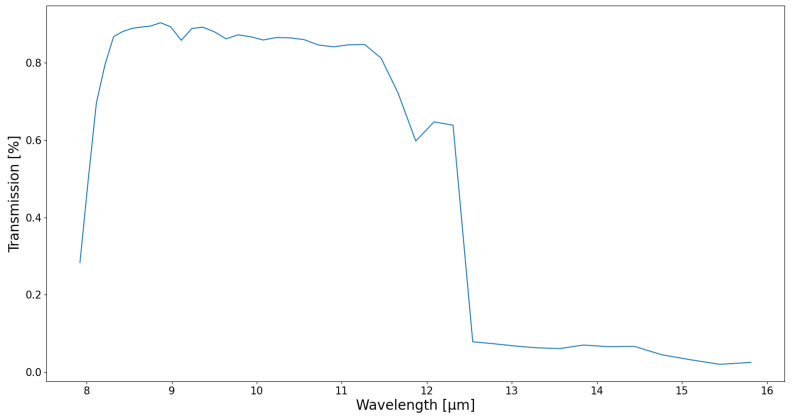
Spectral response of the full optical system, consisting of the germanium lenses and the detector.

For each scene, the narrowband temperature can be computed by applying a similar method to the simulated filtered spectral irradiances, by using Equations (5)–(9). The narrowband irradiance $I_{\mathrm{narrowband}}$ is defined as the integral over the wavelengths of the filtered spectral irradiances $I_{\mathrm{filtered}}\left(\lambda \right)$, which are the product of the spectral irradiances by the spectral response SR(λ). The spectral response SR(λ) takes into account the transmission of the optical system, i.e., the transmission of the germanium material, and the spectral response of the detector (Figure 9).

(5) $$I_{\mathrm{filtered}}\left(\lambda \right)=\mathrm{SR}\left(\lambda \right)I\left(\lambda \right)$$

(6) $$I_{\mathrm{narrowband}}=\int_{\lambda }I_{\mathrm{filtered}}\left(\lambda \right)d\lambda$$

Considering a blackbody at temperature *T*, the narrowband irradiance is a function of this temperature.

(7) $$I_{\mathrm{narrowband}}=I_{\mathrm{narrowband},\mathrm{blackbody}}\left(T\right)$$

**Figure 10:**
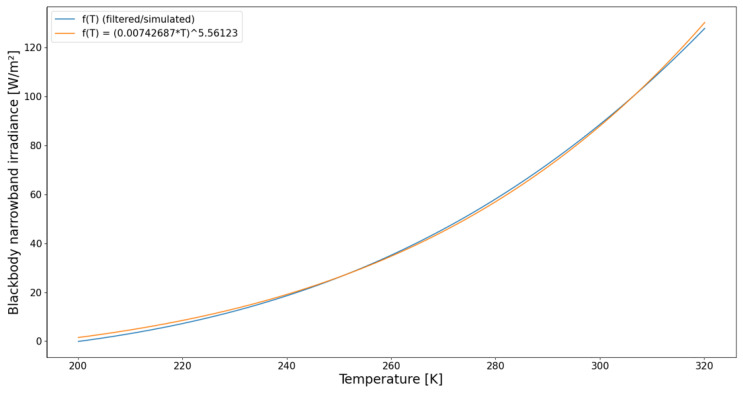
Blackbody narrowband irradiance as function of the blackbody temperature: blue curve = f(T) (filtered/simulated), orange curve = analytical fit.

$I_{\mathrm{narrowband},\mathrm{blackbody}}\left(T\right)$ is illustrated by the blue curve in Figure 10. The analytical fit is illustrated by the orange curve in Figure 10, and is described by Equation (8).

(8) $$I_{\mathrm{narrowband},\mathrm{blackbody}}\left(T\right)\approx {\left(0.00742687\;T\right)}^{5.56123}$$

For an arbitrary scene, the narrowband brightness temperature $T_{\mathrm{narrowband}}$ is defined as the temperature of the equivalent blackbody that results in the same narrowband irradiance $I_{\mathrm{narrowband}}$.

(9) $$T_{\mathrm{narrowband}}=\frac{I_{\mathrm{narrowband}}^{-5.56123}}{0.00742687}$$

### 3.5. Fit of the Broadband Temperature as Function of the Narrowband Temperature

**Figure 11:**
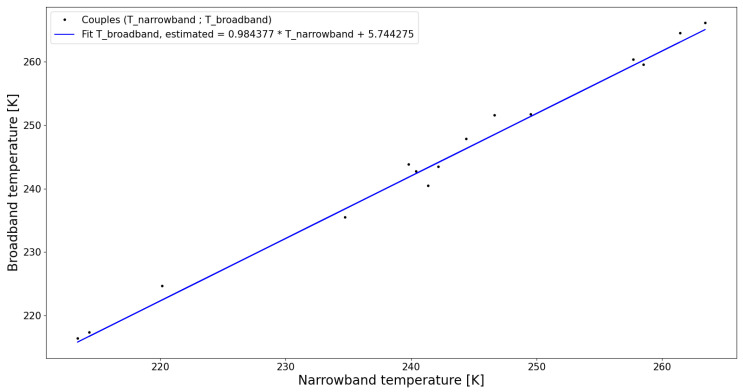
Couples of narrowband temperature and broadband temperature for simulated scenes, listed in Table 3. Line: linear fit of the broadband temperature as function of the narrowband temperature. RMSE = 1.498 and R^2^ = 0.991.

Once the broadband temperature $T_{\mathrm{broadband}}$ and the narrowband temperature $T_{\mathrm{narrowband}}$ are obtained for each scene, we plot $T_{\mathrm{broadband}}$ as function of $T_{\mathrm{narrowband}}$ (Figure 11).

Given the simulated narrowband temperature $T_{\mathrm{narrowband}}$, the broadband temperature can be estimated by

(10) $$T_{\mathrm{broadband}}\approx T_{\mathrm{broadband},\mathrm{estimated}}=0.984377\cdot T_{\mathrm{narrowband}}+5.744275$$

### 3.6. Estimation of the OLR and Calculation of Error on OLR

The OLR can then be estimated by

(11) $$\mathrm{OLR}\approx {\mathrm{OLR}}_{\mathrm{estimated}}=\sigma T_{\mathrm{broadband},\mathrm{estimated}}^{4}$$

Based on Equations (5), (6), (9)–(11), the OLR is estimated from the narrowband temperature and compared to the theoretical OLR, for each of the simulated scenes that were listed in Table 3.

The relative errors on the OLR estimates are calculated using Equation (12) and are given in the last column of Table 4.

(12) $$\Delta \mathrm{OLR}/\mathrm{OLR}\left[\%\right]=\left(\mathrm{OLR}-{\mathrm{OLR}}_{\mathrm{estimated}}\right)/\mathrm{OLR}$$

We conclude that the largest relative error on the OLR equals 4.8% (Table 4), which is within the 5% requirement that was targeted [8].

## 4. Discussion

In order to make a better assessment of the radiative energy fluxes at the top-of-atmosphere, we develop a suite of wide-field-of-view space-based instruments. In addition to the previously published radiometer [8] and shortwave camera [12], providing for the monitoring of the Earth’s total outgoing radiation and Reflected Solar Radiation respectively, in this paper we present the design of a longwave camera to monitor the Outgoing Longwave Radiation. The shortwave and longwave cameras are intended to complement each other by observing the spatial distribution of the reflected radiation and emitted thermal radiation, respectively. Both cameras are featuring the same field of view of 140° and a spatial resolution of minimally 5 km, but the longwave camera is optimized for use in the thermal wavelength range covering 8 to 14 µm. Despite the fact that the shortwave and longwave camera target similar specifications, the shift towards the thermal wavelength range requires however a full redesign of the camera system due to the use of other lens materials, and the wavelength dependency of the focal length and chromatic lens aberrations.

We target a space mission in the so-called morning orbit, providing continuity after the end of life of the Terra mission, which carries the CERES instrument in the morning orbit. Together with the Libera mission that is going to follow the Aqua mission in the afternoon orbit [6], our targeted space mission will provide for a better sampling of the diurnal cycle. To reduce the cost and time of our satellite, we will make use of a 6U CubeSat platform, where 1U will be allocated to our longwave camera.

We assessed the ability to estimate the Outgoing Longwave Radiation from the camera spectral measurements using radiative transfer simulations. As for any simulation of course, all radiative transfer equation (RTE) solvers involve some approximations. Therefore, solutions provided by libRadtran have some uncertainties that are relative to the solution method, especially in the longwave regime [23]. However, we use the DISORT solver, which is one of the most common and accurate RTE solvers of libRadtran, as it is suited for most applications. This has been validated in many international model intercomparison studies for radiance calculations [24], giving confidence in the results generated by this model. Consequently, the spectral brightness temperatures, radiances and irradiances have been simulated for different reference scenes using libRadtran [20]. We derived the narrowband and broadband temperatures for each scene, followed by an estimation of the broadband radiation and quantification of the uncertainty on our estimates. According to these radiative transfer simulations, the estimated stand-alone accuracy of the Outgoing Longwave Radiation estimate from the longwave camera equals 4.8%, which is within the 5% requirement.

Our longwave camera design consists of three germanium singlet lenses. The full field of view equals 140°, enabling to observe the Earth from limb to limb. The targeted spatial resolution of 5 km at nadir is clearly reached since our spot diagram indicated RMS spot radii smaller than the Airy disk for all wavelengths and all fields. Using 768 × 768 pixels with a size of 17 µm of the ULIS/Lynred Pico1024Gen2 detector, we find a nominal spatial resolution of 4.455 km at nadir, beating the requirement of 5 km. Barrel distortion appears to be the main aberration limiting the spatial resolution. The distortion fθ is maximal at 70°, where it equals 18.85%. An accurate assessment of the optical performance is obtained by considering the polychromatic diffraction MTF, which is well suited to assess the image quality. The results show that the polychromatic MTF is at least 0.5 for all fields at 15 cycles per mm, ensuring a good performance of the optical design. At all wavelengths and all fields, the optical design is close to diffraction-limited. Consequently, we have achieved a compact and WFOV optical design, providing for a good resolution in the thermal range between 8 and 14 µm. This wavelength range is relatively broad for a WFOV camera, which is beneficial for the estimation of the broadband radiation that is the Outgoing Longwave Radiation.

Our future work will involve a full tolerancing analysis in view of fabricating a demonstrator and prototype system. The lenses of the optical system will be in-house manufactured using the ultra-precision diamond tooling machine. After the manufacturing of the lenses, each lens surface will first be characterized in the cleanroom using the white light interferometer to evaluate the surface roughness, and a coordinate measurement machine will be used to check the surface shape. Following that, we can mount the design in the laboratory, where it will then be tested and calibrated, in view of the development of a flight model to be integrated on board of a remote sensing satellite for the monitoring of the Earth’s radiation budget.

## 5. Conclusions

We propose to monitor the Earth’s radiation budget with a suite of compact space-based instruments, adequate for integration within a nano- or micro-satellite. These instruments are a wide-field-of-view radiometer, a shortwave camera and a longwave camera, of which the latter one is the subject of this paper.

The ray tracing simulations supplemented with the radiative transfer computations reveal that our longwave camera yields a sufficiently good image quality to enable scene identification with a spatial resolution better than 5 km, while featuring broadband estimation of the Outgoing Longwave Radiation with a relative uncertainty of less than 5%, owing to its large bandwidth.

## Figures and Tables

**Table 1:** Lens data: surface types, materials, thicknesses and diameters. The 3 lenses are made of Ge.

	Front Surface Type	Back Surface Type	Material	Thickness	Diameter
1st lens	Spherical	Spherical	Ge	4 mm	32.4 mm
2nd lens	Spherical	Aspherical	Ge	6 mm	32.4 mm
3rd lens	Spherical	Spherical	Ge	6 mm	27 mm

**Table 2:** RMS spot sizes for all fields, considering the superposition of all wavelengths.

	Half FOV (°)	RMS Spot Size (µm)	Airy Disk (=17.28 µm)
1st field	0	9.95	diffraction-limited
2nd field	5	10.55	diffraction-limited
3rd field	10	12.06	diffraction-limited
4th field	15	13.88	diffraction-limited
5th field	20	15.48	diffraction-limited
6th field	30	16.68	diffraction-limited
7th field	40	15.19	diffraction-limited
8th field	45	14.32	diffraction-limited
9th field	50	13.91	diffraction-limited
10th field	60	13.83	diffraction-limited
11th field	65	13.76	diffraction-limited
12th field	70	14.52	diffraction-limited

**Table 3:** Abbreviations for 15 different scenes, simulated in libRadtran.

Name	Abbreviation
U.S. Standard	us
Tropical	tr
Midlatitude Summer	ms
Midlatitude Winter	mw
Subarctic Summer	ss
Subarctic Winter	sw
U.S. Standard with water clouds	us_wc
U.S. Standard with thin ice clouds	us_ic_thin
U.S. Standard with thick ice clouds	us_ic_thick
Midlatitude Winter with water clouds	mw_wc
Midlatitude Winter with thin ice clouds	mw_ic_thin
Midlatitude Winter with thick ice clouds	mw_ic_thick
Subarctic Summer with water clouds	ss_wc
Subarctic Summer with thin ice clouds	ss_ic_thin
Subarctic Summer with thick ice clouds	ss_ic_thick

**Table 4:** 15 scenes simulated in libRadtran, listed in Table 3, with their broadband and narrowband temperatures, estimates of the OLR and relative errors on these estimates.

Scene	*T*_broadband_ [K]	*T*_narrowband_ [K]	OLR [W/m^2^]	$\frac{\Delta \mathrm{OLR}}{\mathrm{OLR}}$ [%]
U.S. Standard (us)	258.51	259.56	260.21	−1.01
Tropical (tr)	263.44	266.11	265.06	1.56
Midlatitude Summer (ms)	261.41	264.52	263.07	2.16
Midlatitude Winter (mw)	249.53	251.73	251.37	2.16
Subarctic Summer (ss)	257.70	260.34	259.42	1.41
Subarctic Winter (sw)	240.38	242.71	242.36	0.57
U.S. Standard with water clouds (us_wc)	244.39	247.86	246.31	2.47
U.S. Standard with thin ice clouds (us_ic_thin)	241.33	240.46	243.30	**−4.80**
U.S. Standard with thick ice clouds (us_ic_thick)	213.40	216.41	215.81	1.10
Midlatitude Winter with water clouds (mw_wc)	239.79	243.81	241.79	3.28
Midlatitude Winter with thin ice clouds (mw_ic_thin)	234.73	235.49	236.81	2.25
Midlatitude Winter with thick ice clouds (mw_ic_thick)	214.35	217.35	216.75	1.10
Subarctic Summer with water clouds (ss_wc)	246.63	251.60	248.52	**4.80**
Subarctic Summer with thin ice clouds (ss_ic_thin)	242.16	243.47	244.12	−1.08
Subarctic Summer with thick ice clouds (ss_ic_thick)	220.15	224.67	222.46	3.89

## Abbreviations

The following abbreviations are used in this manuscript:

1U	1 CubeSat Unit
ADM	Angular Distribution Models
CERES	Clouds and the Earth’s Radiant Energy System
COTS	Commercial Off-The-Shelf
EEI	Earth’s Energy Imbalance
ERB	Earth’s Radiation Budget
FOV	Field Of View
IMA	IMAge
LW	LongWave
MTF	Modulation Transfer Function
NASA	National Aeronautics and Space Administration
OBJ	OBJect
OLR	Outgoing Longwave Radiation
RMS	Root Mean Square
RMSE	Root-Mean-Square Error
RSR	Reflected Solar Radiation
RTE	Radiative Transfer Equation
SIMBA	Sun-earth IMBAlance
SW	ShortWave
TOA	Top-Of-Atmosphere
TRMM	Tropical Rainfall Measuring Mission
WFOV	Wide-Field-Of-View
